# TAVR Interventions and Coronary Access: How to Prevent Coronary Occlusion

**DOI:** 10.3390/life13071605

**Published:** 2023-07-21

**Authors:** Flavius-Alexandru Gherasie, Alexandru Achim

**Affiliations:** 1 Department of Cardiology, University of Medicine and Pharmacy “Carol Davila”, 050474 Bucharest, Romania; 2 Department of Cardiology, Medizinische Universitätsklinik, Kantonsspital Baselland, Rheinstrasse 26, 4410 Liestal, Switzerland; alexandru.achim@ksbl.ch

**Keywords:** coronary artery obstruction, transcatheter aortic valve replacement, valve-in-valve interventions

## Abstract

Due to technological advancements during the past 20 years, transcatheter aortic valve replacements (TAVRs) have significantly improved the treatment of symptomatic and severe aortic stenosis, significantly improving patient outcomes. The continuous evolution of transcatheter valve models, refined imaging planning for enhanced accuracy, and the growing expertise of technicians have collectively contributed to increased safety and procedural success over time. These notable advancements have expanded the scope of TAVR to include patients with lower risk profiles as it has consistently demonstrated more favorable outcomes than surgical aortic valve replacement (SAVR). As the field progresses, coronary angiography is anticipated to become increasingly prevalent among patients who have previously undergone TAVR, particularly in younger cohorts. It is worth noting that aortic stenosis is often associated with coronary artery disease. While the task of re-accessing coronary artery access following TAVR is challenging, it is generally feasible. In the context of valve-in-valve procedures, several crucial factors must be carefully considered to optimize coronary re-access. To obtain successful coronary re-access, it is essential to align the prosthesis with the native coronary ostia. As part of preventive measures, strategies have been developed to safeguard against coronary obstruction during TAVR. One such approach involves placing wires and non-deployed coronary balloons or scaffolds inside an at-risk coronary artery, a procedure known as chimney stenting. Additionally, the bioprosthetic or native aortic scallops intentional laceration to prevent iatrogenic coronary artery obstruction (BASILICA) procedure offers an effective and safer alternative to prevent coronary artery obstructions. The key objective of our study was to evaluate the techniques and procedures employed to achieve commissural alignment in TAVR, as well as to assess the efficacy and measure the impact on coronary re-access in valve-in-valve procedures.

## 1. Introduction

Since 2002, transcatheter aortic valve replacement (TAVR) has become an increasingly popular and less invasive option for treating symptomatic severe aortic stenosis (AS) compared with conventional surgical aortic valve replacement (SAVR) [1]. Compared with SAVR, TAVR offers equal or superior patient outcomes regardless of risk. Moreover, it has been demonstrated to be cost-effective, providing excellent clinical outcomes and improved quality of life in patients with severe AS. As a result, TAVR has become the preferred intervention method for patients with symptomatic severe AS who are not suitable for standard SAVR [2,3,4,5,6]. It is essential to note that while surgical aortic valve replacement has been the cornerstone of care for severe symptomatic aortic stenosis for a long time, TAVR has now become the predominant treatment in the United States and Europe [7,8,9].

There is a high probability of simultaneously occurring coronary artery disease (CAD) in severe AS, accounting for approximately half of all patients undergoing TAVR. However, the rate has decreased as TAVR is increasingly performed on healthier patients [10,11]. When deciding whether to perform a percutaneous coronary intervention (PCI) before TAVR, it is important to consider integrated management of the case. This includes reducing the risk of ischemic events associated with valve replacement, especially when rapid pacing is needed, and ensuring that the coronary intervention can be easily performed without interference from the valve prosthesis.

Lateef et al. concluded that PCI for severe CAD before TAVR is not clinically advantageous [12,13]. Rather, it seems that patients with severe coronary artery disease who undergo TAVR may experience better outcomes if they undergo complete revascularization, which is determined by recalculating the SYNTAX scores after the procedure [14]. A recent European clinical consensus statement recommends that when considering the possibility of performing PCI after TAVR, it is crucial to select the appropriate transcatheter heart valve, self-expanding valve, or balloon-expandable valve and employ an implantation technique that prioritizes the preservation of unhindered coronary access. The same consensus states that although the available evidence is limited, it generally does not endorse the routine use of PCI before TAVR in asymptomatic lesions. Instead, the evidence indicates that a specific stepwise procedure performed prior to TAVR is a more promising approach when a percutaneous coronary procedure is deemed necessary. Presently, the ongoing COMPLETE TAVR trial (ClinicalTrials.gov: NCT04634240) is in the process of randomizing 4000 patients undergoing TAVR and presenting severe CAD. This encompasses individuals with more than one lesion in a coronary artery, exhibiting more than 70% angiographical diameter stenosis, with a diameter greater than 2.5 mm, while not including chronic total occlusions. The trial aims to compare the outcomes of staged complete revascularization following TAVR with a balloon-expandable valve with the outcomes of solely medical management. The ESC/European Association for Cardio-Thoracic Surgery (EACTS) Clinical Practice Guidelines on Myocardial Revascularization state that patients who have stenosis of over 70% in the proximal segments of their vessels and are scheduled for TAVR should consider coronary angioplasty. This guideline is in effect immediately and provides patients with a clear course of action for optimal treatment [15].

As the age and intensity of complications among TAVR patients decrease, they can enjoy longer and more active lives after undergoing valve intervention. As a consequence, patients may require supplementary diagnostic angiographies and angioplasties. In the meantime, patients could evolve newly developed or advanced coronary artery disease. Those who have undergone PCIs in the past may need to undergo inspection and new procedures due to stent restenosis. 

Vilalta et al. reported from a study conducted over two years that acute coronary syndrome following TAVR occurs as frequently as in 10% of cases [16]. Coronary artery re-access may be compromised due to the anatomical alignment between the coronary ostia and prosthetic aortic valve. When planning a strategy for engaging the coronary arteries, it is important to consider the various design characteristics of the type of transcatheter aortic valves. Several studies have demonstrated the feasibility of coronary angiography and intervention following TAVR [17,18,19,20]. Even so, consistent reports of low success rates have been reported following the insertion of the Medtronic CoreValve (Medtronic Inc., Minneapolis, MN, USA), particularly in the right coronary artery [20,21,22]. An overview of valve deployment strategies is presented in this paper with particular emphasis on valve-in-valve procedures and catheter options to facilitate effective post-TAVR assessments and coronary angioplasties.

## 2. Overview of Strategies to Prevent Coronary Obstruction

Transcatheter aortic valve replacement is a way to replace a damaged aortic valve without undergoing surgery. It can be used to replace either native leaflets or bioprosthetic leaflets. During heart valve implantation, aortic leaflets can be displaced and impede coronary arteries in 0.7% of all cases, necessitating urgent percutaneous coronary interventions or coronary artery bypass grafts [23]. As such, it is important to avoid displacement of the aortic leaflets during implantation. Various techniques and tools can be used to ensure that the aortic valve is correctly placed and that the leaflets are not displaced. Additionally, TAVR valve designs that allow aortic leaflets to be positioned away from the coronary ostia, as well as those that allow the recapture of the leaflets, are being developed to reduce the risk of coronary artery obstruction. In summary, the careful planning and implementation of the TAVR procedure are essential to reduce the risk of aortic valve displacement into the coronary arteries. According to recently published studies, the valve-in-valve TAVR risk is higher (2.3%) [24]. 

The potential for obstruction of one or both of the major coronary arteries due to the coverage of the ostia due to very large native leaflets having been displaced from their positions by the body of the expandable heart valve is a very concerning issue. The leaflets’ displacement results from the frame of the valve covering the ostia, blocking the native flaps from occupying their regular positions. This can result in the narrowing of the coronary artery, which can reduce and even completely obstruct blood flow to the heart. An obstruction of this nature has the potential to give rise to a variety of serious and potentially fatal cardiovascular complications, emphasizing the importance of recognizing the risks connected with this matter. It is important to be aware of the potential for the displacement of native leaflets due to the frame of the THV (transcatheter heart valve), particularly in bicuspid aortic valve interventions, and take the necessary steps to minimize this risk [25]. 

Valve-in-valve (VIV) procedures have become increasingly popular for treating patients with failing bioprosthetic valves. However, these procedures must be performed with caution, as the displacement of the bioprosthetic leaflets that cover the coronary ostia can lead to reduced coronary blood flow toward the sinus of Valsalva (SOV). This occurs if the displaced leaflets, both native and prosthetic, are pushed into contact with the sinotubular junction. Therefore, it is important to pay attention to the displacement of the leaflets and ensure that there is no contact between the leaflets and the sinotubular junction during VIV procedures. The skirt or commissural posts of transcatheter heart valves can obstruct coronary ostia. Less common causes include coronary dissection, hematomas, or embolizations caused by thrombotic or degenerative conditions. It has been reported that the 30-day mortality rate for acute CAO (coronary artery occlusion) during TAVR is high. During TAVR procedures for native aortic stenosis, the complication rate can vary from 8% to 41%. For valve-in-valve procedures, the complication rate can reach up to 53% [24,26].

Some factors can predict coronary occlusion during TAVR procedures, such as being female, having a short sinus of Valsalva, and having a distance from the valve to the coronary ostia that is less than 10 mm (Table 1) [27].

For the accurate measurement of aortic root dimensions, including the distance of the bioprosthetic valve from the coronary artery (VTC), CT angiography is essential. Dvir et al. suggested that having a final VTC size of 3 mm or less carries an increased risk of ostial occlusion [28]. The primary underlying factor in aortic valve-in-valve procedures is the coronary ostia’s proximity to the expected placement of the shifted bioprosthetic leaflets following valve deployment. As a result, conditions that increase the likelihood of CAO may encompass a bioprosthetic valve positioned above the annulus, a sinotubular junction that is both narrow and positioned low, leaflets that are bulky in nature, and coronaries located in a narrow aortic root or those that have been re-implanted.

## 3. Commissural Alignment

Optimal transcatheter aortic valve function and coronary access are associated with commissural alignment. To achieve this, certain factors must be considered. Firstly, the orientation of the valve must be taken into account, as it directly affects the degree of commissural alignment. Secondly, the size and shape of the valve must be taken into consideration, as these can affect the alignment of the commissure. Thirdly, the depth at which the valve is to be inserted must be measured accurately, as too deep an insertion can cause an improper commissural alignment. Finally, the amount of force applied during the insertion must be monitored, as too much force can lead to improper commissural alignment. All these factors must be taken into account to ensure optimal coronary access through a proper commissural alignment [29].

To achieve optimal fluoroscopic angulation, pre-procedural MDCT (multidetector computed tomography) calculates the annular plane of the aortic valve based on the cusp hinge points. Multiple angulations are visualized across the S-curve of the valve until the designated hinge points for the LCC and RCC overlap, thus isolating the NCC on the opposite side. The cusp-overlap angulation is defined using the fluoroscopic angulation [30].

The right–left cusp overlap view is the preferred method for the commissural alignment of a transcatheter aortic valve. This view isolates the commissure, which is the 120-degree separation along the right and left cusps on the right of the fluoroscopic view. This view is important as it allows for a more accurate alignment of the THV, ensuring that it is properly secured in the aortic annulus. It is also ideal for the accurate sizing of the THV, as the commissural alignment can help to determine the optimal size of the valve. Proper alignment is essential for a successful THV procedure, and this view is an essential part of the process. The left–right cusp overlap view is an important imaging technique for assessing coronary access. For ideal commissural alignment, the left and right coronary arteries must emerge from the left–right commissure at an angle of 60 degrees. If the coronary arteries emerge at an angle smaller or larger than 60 degrees, this could indicate a misalignment. It is important to recognize misalignment, as it can indicate coronary artery disease or other vascular issues. The left and right coronary arteries must emerge from the left–right commissure at the correct angle to ensure that the best possible coronary access is achieved [31,32].

In cases where commissural alignment leads to coronary misalignment as a result of coronary ostium eccentricity, coronary alignment is recommended. In order to line up the nadir of the bioprosthetic leaflets with the coronary ostia in these cases, the operator must calculate an alternative THV rotation angle to preserve a 60-degree angle from the TAV commissural post and the coronary ostia [33,34]. 

Based on the method of deployment, TAVR devices can be categorized as either balloon-expandable or self-expandable (Table 2).

Our next section focuses on the disparities in commissural alignment among the three major TAVR platforms.

### 3.1. CoreValve Evolut R and PRO Valves (Medtronic Minneapolis, MN, USA)

The CoreValve Evolut R and Pro devices are equipped with three commissural frame posts, each measuring 26 mm in height. One of these posts is positioned in line with the paddle on the C-Table. One way to predict the direction of the commissural alignment for the ostium is by observing the hat’s orientation on the delivery catheter during deployment. This is due to the fact that the hat on the delivery catheter is always oriented in the same direction as the commissural frame posts, making it easy to identify the orientation of the coronary ostia.

A new study discovered that if the hat indicator of the CoreValve Evolut delivery system is aligned with the aortic curve and center front of the aorta on the coplanar three-cusp angiographic view, it can significantly reduce the incidence of severe coronary overlap. Out of all the positions, this one had the least amount of overlap, at only 23.2%. However, when the hat marker is oriented toward the inner curve and center back of the aorta, the overlap frequency increased to 75%. This finding is crucial as it helps to identify the most optimal system positioning for better overall patient outcomes. By directing the hat marker along the inside curve and center back of the aorta, the frequency of overlap increased to 75%. This discovery is critical in identifying the optimal system positioning for better patient outcomes [35].

For patients with severe AS who also have other conditions like chronic or acute coronary syndromes that require easy access to coronary arteries for additional coronary angiography, optimizing the alignment of the TAV commissures is important. This is especially crucial for younger patients who are at lower risk. For the CoreValve Evolut R/Pro implantation system, it is recommended to introduce it through the common femoral artery using flush ports at the 3 o’clock position. The hat marker is oriented to face the aortic root’s outer curve when the delivery system passes through the aortic valve. This ensures proper commissural alignment and prevents overlap with the coronary ostia [36].

### 3.2. SAPIEN XT and SAPIEN 3 Valves (Edwards Lifesci-Ences, Irvine, California)

SAPIEN 3/Ultra is the only transcatheter heart valve authorized in Europe and the United States for a second TAVR procedure. Recently, an operative guidance consensus was published on procedural planning and techniques for this particular valve [37]. Compared with the CoreValve Evolut, the Sapien 3 has a 3 mm tab positioned on the commissural primary rows instead of a commissural post. Even though the Sapien 3 device has a lower frame height than the Evolut device, the commissural tabs may find themselves next to the coronary ostium, particularly if the coronary origin has a low origin. Crimping the Sapien 3 valve at different angles prevents the possibility of intentional commissural alignment. This is because the angle of the crimp has a direct effect on the alignment of the commissures. If the crimp is not consistent, the alignment of the commissures is affected, making it difficult to achieve an intentional alignment [38]. Compared with the Evolut device, which contains a supra-annular valve system, Sapiens 3 valves are balloon-expandable intra-annular valves, giving them an advantage in coronary occlusion prevention. Lopes et al. presented a report regarding the failure of a 34 mm Evolut Pro valve implantation, which led to coronary occlusion and cardiac arrest. The quick recognition of acute left main occlusion caused by the high valve implantation resulted in the immediate initiation of advanced life support. The valve was quickly pulled toward the ascending aorta utilizing the snare technique, which immediately restored the flow and facilitated effective cardiopulmonary resuscitation. Following this, a 29 mm balloon-expandable Sapiens 3 valve was implanted successfully [39]. In general, the position of the second valve should be chosen to improve the procedure’s results and reduce the risk of coronary occlusion and sinus compression. It is important to implant the second valve at a low implanting depth to reduce the risk of coronary blockage, enabling various levels of overlap between the valve’s leaflets. Before proceeding with the index transcatheter heart valve intervention, it is important to evaluate the alignment of the neo-commissure. This determines the technique’s effectiveness, as significant misalignment during the first valve procedure could limit its benefits.

### 3.3. Acurate Neo Valve (Boston Scientific, Marlborough, MA, USA)

Accurate Neo is a self-expanding transcatheter valve with a supra-annular configuration and porcine pericardial leaflets available in Europe since 2014. The top-down positioning facilitates optimal positioning and limits flow obstruction upon deployment. A total of three stabilization pillars facilitate better coaxial alignment, and the superior crown enhances anchoring. As long as the upper crown keeps the native cusps away from the coronary ostia, there is a low risk of coronary obstruction. 

The coronary re-access procedure is made less challenging by the top crown’s short stent design and open-cell architecture. This design allows for easier and faster deployment, as well as improved stent visibility. Additionally, the open-cell design of the upper crown makes it easier to pass a guidewire distally, if needed. This makes the procedure easier and more efficient, resulting in shorter procedure times. Furthermore, the short stent profile and open-cell configuration of the top crown make it easier to access the distal vessel, which can be beneficial in certain cases. SAVI TF registry records report that there have been no cases of coronary obstruction that require intervention in 1000 patients [40]. 

Vanhaverbeke et al. reported a successful valve-in-valve replacement with a Sapiens valve in a degenerated ACURATE Neo prosthesis [41]. A typical THV is equipped with a complete frame, which allows the leaflet to form the neoskirt. The neoskirt of the ACURATE neo does not extend to the upper crown of the SAPIEN 3, which minimizes the risk of coronary obstruction and promotes optimal coronary access. ACURATE Neo also has the advantage of not overexpanding when it performs TAV-in-TAV. To confirm these encouraging observations, more SAPIEN-in-ACURATE cases in the real world are needed, despite the extensive modeling and testing that has been pre-procedurally conducted on the current TAV-in-TAV case and the clinical outcomes confirming both bench tests and computational models.

## 4. Angiograms and Interventions following TAVR

Understanding the location of the coronary arteries relative to the THV is the first step in the angiographic imaging of coronary arteries. When performing coronary catheterization, the catheter used is often chosen based on the shape and design of the transcatheter heart valve. Typically, the left main coronary artery and right coronary artery origins are seen in a left anterior oblique (LAO) projection.

For CoreValve Evolut coronary re-access, the standard JR4 or JL4 diagnostic catheters are generally the most suitable options. However, in some cases, a half-size smaller JL catheter may be preferable [42,43]. This is because it can provide a more comfortable fit while still offering the same degree of accuracy as larger catheters. Additionally, the smaller catheter may also be easier to maneuver in smaller lumens or vessels. In any case, it is important to consider the individual patient’s anatomy and preferences when selecting a catheter. Ultimately, the choice of the right catheter helps ensure a successful re-access procedure. Although various catheters may be able to provide results, Ikari efficiently delivers for both coronary arteries. To perform sub-selective angiography, any cell above the coronary artery can be engaged. If selective imaging must be performed, inserting a wire into the vessel and guiding the catheter with the wire to introduce the catheter is extremely useful.

Edwards Sapien 3 valves are often placed above the coronary ostia, their frame is taller, and the upper cells are more prominent, resulting in less interference with coronary ostia access. It is uncommon to experience coronary obstruction when using the “high implant-90/10” implantation technique, which helps to minimize interference with the left ventricular outflow tract. In most cases, a specific catheter selection does not need to engage the coronary arteries with a Sapien 3 valve. To avoid altering the position of the Sapien 3 valve or managing the task of disengaging the guiding wire post-intervention, a guide extender is recommended for percutaneous coronary interventions [44,45]. 

With the bench-testing of patient-specific 3D-printed models, it has been demonstrated that diagnostic angiography and PCI are highly feasible following ViV-TAVR using the ACURATE neo valve [46]. It was reported that 62/64 (97%) cannulations were suitable for the assessment of angiography. On average, only one attempt was needed for cannulation, and the procedure took approximately 1 min and 48 s to complete. Selective cannulation was achieved in 82% of cases (51 out of 62). As a rule, in most of the situations, no more than one cannulation attempt was needed (49/62, 79%). A total of 9 out of 62 (15%) cannulations involved complex cannulation techniques with 0.014” coronary wire-assisted cannulation being the most frequently used approach.

In terms of cannulation feasibility, selectivity, attempts, time, or technique, no significant disparities were noted between the LCA and RCA. It was viable to conduct the entire PCI procedure in 61/64 (95% of cases).

## 5. Basilica Procedure

**Figure 1:**
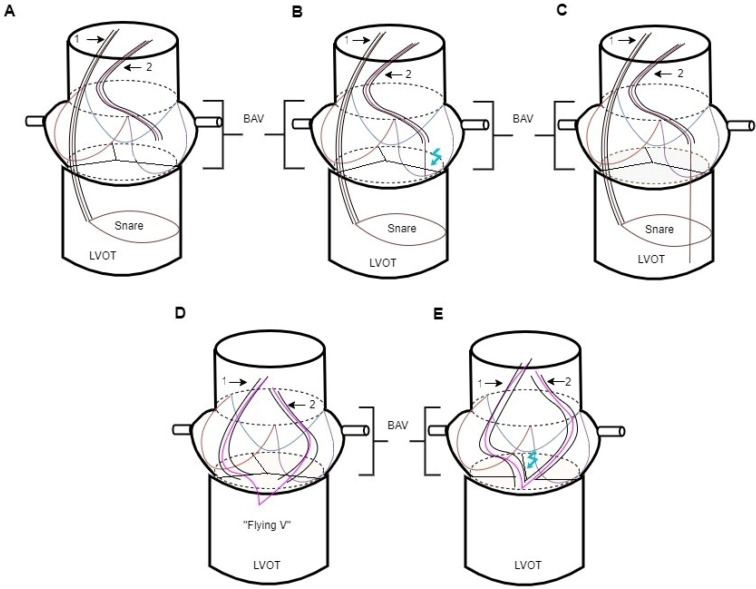
BASILICA procedure on the left aortic leaflet in a biological aortic valve. 1—MP1; 2—AL2. Steps: Amplatz Left 2 (AL2) guiding catheter is placed in the ascending aorta. Goose Neck snares are delivered into the LVOT through a Multipurpose 1 (MP1) guiding catheter (**A**). After the placement of an 0.014″ Astato wire into the AL2 until it reaches the lowest point of the left aortic leaflet, it is electrified before crossing over into the LVOT. (**B**). The 0.014″ Astato wire is snared (**C**). In the next step, the snare is externalized, and the “flying V” is placed (**D**). Injection of 5% dextrose water into both catheters results in laceration of the leaflet with the Astato wire, causing it to splay outward (**E**). LVOT, left ventricular outflow tract; BAV, biological aortic valve.

During the valve deployment procedure, leaflets may be lacerated, or the coronary ostia may be protected with a guide catheter to prevent obstruction. Before a transcatheter heart valve implantation, a BASILICA (bioprosthetic or native aortic scallop intentional laceration to prevent iatrogenic coronary artery obstruction) procedure uses a transcatheter electrosurgical process. This involves crossing the aortic leaflet and lacerating it in line with the exposed coronary artery ostium to ensure blood flow to the coronary arteries is maintained after the valve implantation, thus avoiding coronary occlusion (Figure 1) [47,48].

During such a procedure, a wire loop is created by snagging the guidewire within the left ventricle. A non-insulated, non-coated portion of the wire is positioned at the leaflet to split the aortic leaflet, and electricity is applied. To reduce coronary obstruction following the valve implantation, the bioprosthetic leaflet or native leaflet is separated. It may be more challenging to lacerate leaflets with excessive calcification or thickening. 

A prospective, multicenter registry named BASILICA tracks patients at risk for iatrogenic coronary occlusion following TAVR using the BASILICA technique. In North America and Europe, the registry enrolled 214 patients. 72.8 percent of patients had bioprosthetic aortic valves. There was a success rate of 94.9% and 94.4% for leaflet traversal and laceration, and the prevalence of coronary obstructions (partial or complete) was 4.7% [49,50,51].

In Europe, EURO-BASILICA provides the first multicenter study investigating the BASILICA technique. A one-year clinical study demonstrated that this technique was effective and viable in protecting against CAO caused by TAVR. There were 76 patients enrolled across ten centers in Europe, 5.3% of whom had native aortic valves, 92.1% had surgical bioprosthetic valves, and 2.6% had transcatheter valves. The proportion of patients who required double BASILICA (for both coronary cusps) was 11.8%. The BASILICA approach resulted in successful outcomes in 97.7% of cases, with only a small percentage (2.4%) experiencing complete coronary occlusion. Additionally, in 90.6% of situations, there were no instances of leaflet-associated coronary occlusion [52]. A total leaflet-induced occlusion was documented in two patients. In one case, the left coronary artery was occluded, and extracorporeal membrane oxygenation was needed for one hour. Using the chimney technique, the occlusion was treated with ostial stenting. In the second case, the left coronary artery ostium was completely obliterated by the partial avulsion of a bioprosthetic valve leaflet. Ostial stenting was used to treat the obstruction, and Impella was used to provide mechanical support.

## 6. Chimney Stenting Procedure

**Figure 2:**
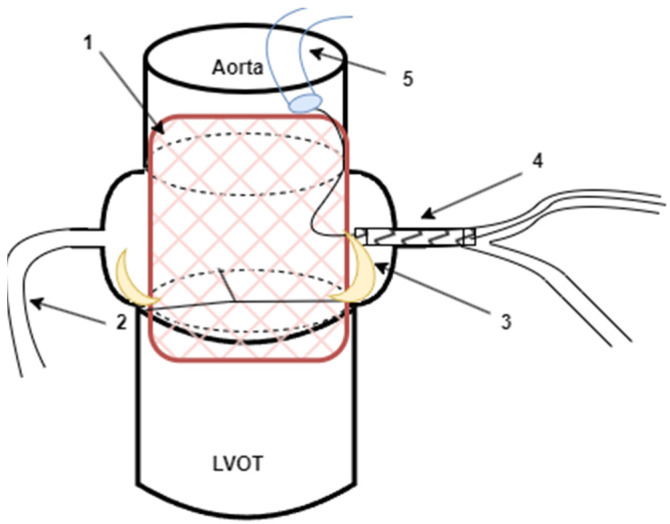
During TAVR implantation, a chimney stenting procedure is performed due to occlusion of the left coronary artery: 1—TAVR valve implanted; 2—the right coronary artery, 3—dislocated leaflet causing CAO; 4—left main stenting as a bailout strategy; and 5—Luncher guide catheter.

Chimney stenting is an acceptable (and less laborious than the BASILICA) bailout technique among patients with established coronary artery occlusion and those without upfront coronary protection. Before expanding the valve, the “chimney” technique involves using a guide catheter and guide extender to engage the coronary artery (Figure 2).

The chimney stenting technique and flow chart is conducted as follows: Step 1, place an adequately dimensioned coronary stent in the mid-left anterior descending coronary artery before the implantation of the transcatheter heart valve. The scaffolding must be long enough to extend into the ascending aorta until it reaches the sinotubular junction. Step 2, when implanting the valve, withdraw the guide catheter and place it in the ascending aorta. Step 3, in case of compromised coronary blood flow, meticulously pull the non-deployed stent back over the coronary ostium and over the dislocated aortic leaflets and then implant it. Step 4, if the valve needs post-dilatation, complete simultaneous kissing balloon inflation with the chimney stent. Step 5, complete a final angiographic check.

Protecting the coronary artery during TAVR typically involves using additional arterial access to engage the guide catheter in the at-risk coronary artery. It is recommended to use Judkins left/right or multipurpose guiding catheters during THV deployment since these catheters allow quicker backup into the ascending aorta and can be moved closer to the ostium after THV deployment. Using other catheters (Ikari, EBU, or AL1) may also be successful. However, they may be more difficult to reposition after THV implantation. As a precaution against hemodynamic instability following balloon aortic valvuloplasty (BAV), a coronary guide catheter needs to be attached and a guidewire placed distally into the vessel. Having positioned a balloon or stent over the wire distally in the artery, the guide catheter is withdrawn slowly into the ascending aorta. The anatomical factors concerning the aortic root and the apprehension of the risk of CAO may influence the judgment to place a balloon or scaffold on the guidewire. Although using only a guide wire can save costs and time, it can be challenging to deliver a stent next to an implanted valve and dislocated native leaflets due to calcium build-up or the protection guidewire becoming stuck [53,54]. The failure rate of stent deployment was 10 to 20 percent in two observational trials in which stenting was attempted for the treatment of CAO during TAVR [26,55].

The International Chimney Registry was founded in October 2017, included 60 patients, and gathered retrospective data from 16 centers in North America, Europe, and the Middle East on individuals experiencing chimney stenting as part of transcatheter aortic valve interventions from May 2010 to July 2018. The database did not include data from cases with initial coronary protection involving no chimney scaffold implantation or patients with coronary artery obstruction resolved using other strategies. Among the arteries stented, the left main was more commonly treated by itself (49 cases; 81.6% of the total) or in conjunction with the right coronary artery (6 patients; 10% of the cases). In most cases (96.6%), drug-eluting stents were used, with an average stent size and diameter of 19 ± 7.8 and 4.1 ± 0.5 mm, according to the data. Many incomplete stent expansions were observed in 55% of cases, requiring further dilation or implantation of a second scaffold within the initially implanted stent (11 patients). A total of 2 patients (3.3%) underwent post-dilatation of the transcatheter heart valve with a kissing balloon technique. In 19 cases with mean gradients of over 20 mm Hg, 90% had experienced a valve-in-valve intervention. The number of patients with more than mild paravalvular leaks was 3 (5.0%). Out of the total number of patients, 3 (5.0%) died while in hospital. One patient was unresponsive after being removed from a cardiopulmonary bypass on the first day, another patient succumbed on the fifth day due to complications related to cardiogenic shock, and the third patient died on the eleventh day from septic shock following the intervention [56,57]. The COPROTAVR registry collected data on 236 patients at risk of coronary occlusion who had undergone transcatheter aortic valve implantation with a coronary safety guidewire in place [58]. Out of the total number of participants in the trial, 143 patients underwent coronary stenting, which makes up 60.6% of the total. Of these patients, 79% received chimney stenting, while 21% received ostial stenting. After a 3-year follow-up, patients who underwent stenting had positive outcomes with lower rates of cardiac mortality (7.8%), myocardial infarction (9.8%), and stroke (5.4%). Although stent thrombosis was infrequent (0.9%), it posed a life-threatening risk in every instance [58].

These trials provide initial indications of reasonably acceptable mid-term safety when it comes to chimney stenting. However, it is important to approach these retrospective findings with caution due to the limited number of patients involved. Consequently, we advise against employing chimney stenting as the primary strategy for younger individuals or those with more severe CAD. Instead, angioplasty should only be considered a rescue alternative in situations where a potential or existing coronary artery obstruction necessitates immediate intervention.

## 7. Conclusions

As part of selecting an appropriate THV, it is important to carefully consider the valve’s shape and how it relates to the coronary arteries. It is also crucial to thoroughly examine the computer tomography study before the procedure to fully understand the anatomy of the coronary ostia, sinus of Valsalva, and sinotubular junction. Numerous factors need to be taken into account to facilitate coronary re-access in valve-in-valve procedures. Identifying the risk factors for coronary artery occlusion during TAVR is crucial, as this is a rare yet grave, life-threatening issue. If the valve neoskirt extends over at least one of the coronaries and the valve–aorta distance is less than 3–4 mm, it is recommended to use a coronary guide wire and a pre-mounted stent. This is particularly important if the additional transcatheter valve heart is planned to be placed high or if there is a risk of the index THV stent expanding excessively. Chimney stenting can be performed in order to restore coronary flow in the case of impending or established CAO. For patients who are at high risk of coronary occlusion, the BASILICA approach may be considered as an option. However, it is important to evaluate the positioning of the first implanted valve with the native aortic valve before the procedure. If there is severe misalignment, the outcome of the technique may be compromised. The long-term effectiveness (>12 months) of both CAO prevention techniques remains unknown.

## Figures and Tables

**Table 1:** Risk factors for coronary occlusion.

VIV	TAVR
Stentless valve design	Female gender
Stented prosthesis with leaflets outside	Coronary height of <10 mm
Distance of the bioprosthetic valve from the coronary artery of <4 mm	Sinus of Valsalva of <28 mm
	Leaflet elongation according to coronary height
	Masses of calcium
	Leaflet dimensions and placement

**Table 2:** Main TAVR devices and characteristics.

JenaValve JenaValve Technology GmbH	Lotus Boston Scientific Corporation	Portico St. Jude Medical, Inc.	Acurate Neo Symetis	Evolut R Medtronic	Sapiens 3 Edwards Lifesciences	Valve Name
Self-expandable porcine pericardial tissue	Self-expandable bovine pericardial tissue	Self-expandable bovine pericardial tissue	Self-expandable porcine pericardial tissue	Self-expandable porcine pericardial tissue	Balloon-expandable bovine pericardial tissue	Structure Type
-	≥6 mm for 23 mm device size ≥6.5 mm for 25 mm and 27 mm device size	≥6 mm	≥6 mm	≥5 mm for 23 mm for 26 mm and 29 mm device size ≥5.5 mm for 34 mm device size	≥5 mm for 23 mm and 26 mm device size ≥5.5 mm for 29 mm device size	Access Vessel Diameter
TA sheathless 32 F (23, 25, and 27 mm)	TF 18 F (23 mm) 20 F (25 and 27 mm)	TF, TAo, TSc 18 F (23 and 25 mm) 19 F (27 and 29 mm)	TF 18 F TA sheathless 28 F	TF, TAo, and TSc 14 F (23, 26, 29, and 34 mm)	TF 14 F (20, 23, and 26 mm), 16 F (26 mm) TA, TAo 18 F (20, 23, and 26 mm), 21 F (26 mm)	Access Type Device Size
Yes	Yes	Yes	No	Yes	No	Repositionable

TF, transfemoral; TA, transapical; TAo, transaortic; TSc, trans-subclavian.

## Data Availability

Not applicable.
